# Beyond the first case of Chagas disease: the Berenice strain as a model for understanding long-term *Trypanosoma cruzi* infection

**DOI:** 10.1590/0074-02760240291

**Published:** 2025-07-21

**Authors:** Diana Bahia, André Guilherme da Costa-Martins, Werica Bernardo Pereira, Fernanda Sycko Marchiano, Camila Miyagui Yonamine, José Franco da Silveira

**Affiliations:** 1 Universidade Federal de Minas Gerais, Instituto de Ciências Biológicas, Departamento de Genética, Ecologia e Evolução, Belo Horizonte, MG, Brasil; 2 Instituto de Pesquisas Tecnológicas, Laboratório de Micromanufatura, São Paulo, SP, Brasil; 3 Universidade Federal de São Paulo, Escola Paulista de Medicina, Departamento de Microbiologia, Imunologia e Parasitologia, São Paulo, SP, Brasil

**Keywords:** Trypanosoma cruzi, Chagas disease, patient Berenice, the first human case of Chagas disease, strains Berenice, genetic variability, long-term chronic infection, inapparent infection, dormant amastigotes, parasite intra-host evolution, adaptive mechanisms

## Abstract

Here, we review the key findings on the genetic characterisation of Berenice strains of *Trypanosoma cruzi* isolated from a 2-year-old child, Berenice, the first patient with Chagas disease described in the literature in 1909. Be-62 and Be-78 strains were isolated from Berenice when she was 55 and 71 years old, respectively. They were comparatively studied, revealing several important genetic differences that indicated the presence of heterogeneous *T. cruzi* populations within the infection of patient Berenice. Recently, a high-quality whole-genome assembly was generated using the strain Be-62, which was isolated in 1962. Even after decades-long persistence in the patient, there is a high level of conservation in synteny between Be-62 and different *T. cruzi* lineages. It has been suggested that *T. cruzi* diversity is driven by the evolution of multigene families encoding target antigens of anti-parasite immune responses, located in disruptive regions of the genome. Most studies of Berenice have been conducted on genomic bulk samples, resulting in a biased analysis that favours the dominant genotype. Single-cell omics technologies enable us to study the genetic diversity within an infection caused by protozoan parasites in detail. Sequencing individual genomes of Berenice strains will be the key to elucidating the population structure of individual infections, the dynamics of parasite populations, and adaptive mechanisms.

**The discovery of Chagas disease and the isolation of the Berenice strain of *Trypanosoma cruzi***

In April 1909, Carlos Chagas discovered the first human case of Chagas disease (CD) while examining Berenice, a two-year-old girl from the countryside of the State of Minas Gerais (southeast Brazil) who displayed the clinical symptoms of the severe acute clinical form of the disease. Berenice lived in a house infested by a haematophagous insect, the triatomine *Panstrongylus megistus*. Chagas identified a new species of the genus *Trypanosoma* in the hindgut of triatomines, which he named *Schizotrypanum cruzi* (later renamed *Trypanosoma cruz*i), in homage to his teacher, Oswaldo Cruz.^1^ ^,^ ^2^ ^,^ ^3^ ^,^ ^4^ In addition to discovering the disease, Chagas identified the etiological agent and its vector, described the domestic and wild cycles of the parasite and the acute and chronic clinical forms.^1^ ^,^ ^2^ ^,^ ^3^ ^,^ ^4^ We also refer to the publications^5^ ^,^ ^6^ ^,^ ^7^ and references therein for more details.

The Fig. 1 depicts the most significant milestones in the history of the Berenice strain after the discovery of Chagas. In April 1961, 53 years after being affected by the acute phase of CD, Berenice underwent a general clinical examination emphasising CD symptomatology by Salgado et al.^8^ Although the clinical symptoms of CD were mild (indeterminate form), the serology for CD and xenodiagnosis were positive. In 1978, blood trypanosomes were again isolated from the patient Berenice.^9^ ^,^ ^10^ Even though the presence of parasites in the peripheral circulation, Berenice had a normal electrocardiogram and no apparent chagasic lesions.^8^ ^,^ ^11^ Berenice might be a record of permanence and survival of *T. cruzi* with no late disease manifestations.^8^ ^,^ ^11^ Since she has continued to live in an endemic region, we cannot completely rule out the occurrence of a second infection.^8^ ^,^ ^11^

**Fig. 1:**
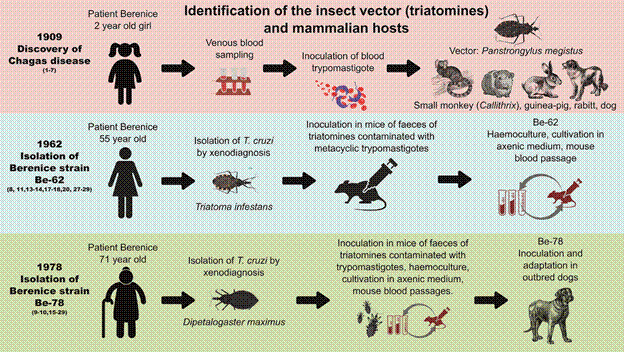
key events in the history of Berenice strains (Be-62 and Be-78) isolated from the first patient diagnosed with Chagas disease (CD). Top (red) panel: In 1909, Carlos Chagas discovered CD in Berenice, a two-year-old girl. He identified the etiological agent *Trypanosoma cruzi*, the insect vector, acute and chronic clinical manifestations, and mapped possible mammalian experimental hosts, describing the complete disease cycle. He also described the parasite morphology in peripheral blood and the insect vector.^1^ ^,^ ^2^ ^,^ ^3^ ^,^ ^4^ ^,^ ^5^ ^,^ ^6^ ^,^ ^7^ Middle (blue) panel: Fifty-three years later, Salgado et al.^8^ re-isolated *T. cruzi* through xenodiagnosis with *Triatoma infestans* when Berenice was 55 years old. They designated this strain Be-62. The first molecular analyses using restriction enzymes and isoenzymes were subsequently performed on this strain.^8^ ^,^ ^11^ ^,^ ^13^ ^,^ ^14^ ^,^ ^17^ ^,^ ^18^ ^,^ ^20^ ^,^ ^27^ ^,^ ^29^ Bottom (green) panel: Sixteen years later, at age 71, Berenice again underwent xenodiagnosis with *Dipetalogaster maximus*. Researchers isolated trypanosomes named Be-78. This isolation allowed researchers to compare both isolates using molecular and genetic analyses.^9^ ^,^ ^10^ ^,^ ^15^ ^,^ ^16^ ^,^ ^17^ ^,^ ^18^ ^,^ ^19^ ^,^ ^20^ ^,^ ^21^ ^,^ ^22^ ^,^ ^23^ ^,^ ^24^ ^,^ ^25^ ^,^ ^26^ ^,^ ^27^ ^,^ ^28^ ^,^ ^29^

In both cases, blood trypanosomes were isolated from patient Berenice by xenodiagnosis using *Triatoma infestans* ^8^ or *Dipetalogaster maximus* ^9^ ^,^ ^10^ (Fig. 1). Triatomine faeces containing metacyclic trypomastigotes were peritoneally inoculated into mice, and successive blood passages maintained the infection. In addition, blood samples containing trypomastigotes were cryopreserved in liquid nitrogen and also isolated by haemoculture and cultivated in axenic medium liver infusion tryptose (LIT), for multiplication of epimastigotes and differentiation into metacyclic trypomastigotes.^8^ ^,^ ^9^ ^,^ ^10^ The isolates were named Berenice Be-62 and Be-78.^10^

Following the exuberant acute phase described by Chagas in 1909 in the patient Berenice,^1^ ^,^ ^2^ the disease progressed to an indeterminate form.^8^ ^,^ ^9^ ^,^ ^10^ Salgado et al.^8^ highlighted that “this case historically documented seems to point at the possibility of human infection for about half a century without producing any known clinical manifestation”.

Like the patient Berenice, many other chagasic patients are asymptomatic despite having positive serology.^7^ ^,^ ^11^ In this context, Garnham^11^ evaluated the role of inapparent infections in the natural history of CD and to what extent the inapparent infections could constitute a reservoir. On that occasion,^11^ he also asked researchers to pay more attention to the behaviour of *T. cruzi* in wild hosts, such as the opossum. Recently, Jansen et al.^12^ reviewed their data on wild reservoirs of *T. cruzi* collected over the past 20 years. *T. cruzi* is transmitted by a wide range of wild mammalian species, where the parasite establishes long-term infections characterised by low parasitaemia levels. They reported that 17% of terrestrial wild mammals were seropositive for *T. cruzi*, and 8% of all samples (n = 6587) showed positive haemocultures, indicating high parasitaemia and infectivity potential.^12^

Nowadays, non-replicating amastigotes, also known as dormant amastigotes, have emerged as an important mechanism for maintaining *T. cruzi* in mammalian hosts. For instance, dormancy has been applied to understand the resistance of *T. cruzi* to trypanotoxic drugs.^13^ What could be the relationship between dormant amastigotes and inapparent infections in *T. cruzi*? Trypomastigotes derived from dormant amastigotes can parasitise new host cells and reestablish new progenies of replicating and non-replicating amastigotes. Trypomastigotes can also reach the host bloodstream and be taken up by blood-feeding vectors.^13^ The host immune system is responsible for recognising and eliminating *T. cruzi*. Host cells carrying dormant amastigotes are not recognised by CD8^+^ T cells and may function as reservoirs for continuous liberation of trypomastigotes.^13^

The isolation of the Be-62 and Be-78 strains remains a unique opportunity to learn about the evolution of the disease and its etiologic agent in the same patient over time, 53 and 69 years after the infection. Some fundamental questions may come up with these initiatives. Would the Be-62 be the same strain isolated from the patient Berenice by Carlos Chagas? The response to this question is still uncertain. Since Berenice lived in the endemic region all the time, it is impossible to rule out the hypothesis that she was re-infected with *T. cruzi*.^8^ ^,^ ^11^ Another possibility might be that Berenice had been infected with triatomines carrying mixed *T. cruzi* populations. This enigma could be solved by examining samples of fixed organs or tissue fragments preserved in paraffin-embedded tissue blocks collected from animals infected with the strain isolated by Carlos Chagas from patient Berenice. If such material still exists, it could be evaluated by molecular “palaeontologists” with DNA probes developed for the *T. cruzi* diagnosis, genotyping and genome sequencing. We will revisit this subject in the following items.

Phenotypic traits of Berenice strains (Be-62 and Be-78)

The phenotypic traits of Be-62 and Be-78 strains were identified in mammalian hosts, mainly in mice and dogs, by parasitological and molecular tools^9^ ^,^ ^10^ ^,^ ^14^ ^-^ ^32^ (Table). Although isolated from the same patient, these strains differ in many phenotypic traits, such as the course of parasitaemia, mortality, morphology of trypomastigotes, cell growth and differentiation, tissue tropism, pathogenicity^9^ ^,^ ^10^ ^,^ ^14^ ^-^ ^29^ and resistance to benznidazole (BZN)^22^ ^,^ ^30^ ^,^ ^31^ ^,^ ^32^ (Table).

**TABLE:** Phenotypic traits of Berenice strains

Phenotypic traits							
Host	Albino mice		Dogs		Dogs		Swiss mice
Parasitaemia	Blood Trypomastigote		Blood Trypomastigote		Metacyclic Trypomastigote		Metacyclic Trypomastigote
Strain	Be-62	Be-78	Be-62	Be-78	Be-62	Be-78	Be-78
Inoculum	150,000 BT^14^ ^,^ ^15^	5,000 BT^9^ ^,^ ^10^ ^,^ ^16^ ^-^ ^20^	2,000 BT/kg body weight^23^ ^,^ ^24^	2,000 BT/kg body weight^16^ ^,^ ^23^ ^,^ ^24^	2,000 MT-triatomine /kg body weight^23^ ^,^ ^24^	2,000 MT-triatomine/kg body weight^23^ ^,^ ^24^ ^,^ ^25^ ^,^ ^26^	5,000 MT-axenic culture^21^
IP	+	+	+	+	+	+	+
CO	-	-	-	-	+	+	-
OR	-	-	-	-	-	-	+
Maximal parasitaemia peak (No. trypomastigotes × 10^6^/mL blood)	1965 2.30^14^ 1964-1972 2.17-4.03^15^	2nd peak 0.62^16^ 1.22^17^ 0.94^18^	2nd peak 0.13^24^	2nd peak 0.02^20^ 0.14^24^ 1.00^25^	2nd peak CO = 5.28; 0.58; 0.28^23^	2nd peak IP = 0.32^24^ CO = 0.49^24^ CO = 1.36; 0.86; 0.33^23^ IP = 1.20^25^	IP = 5.3^21^ OR = 6.3^21^
Patent period (days)	7^14^ ^,^ ^15^	26	15-23^23^ ^,^ ^24^	27-41^16^ ^,^ ^23^ ^,^ ^24^	22-42^24^	29-50^24^	IP = 41^21^ OR = 36^21^
Mortality	100%^14^ ^,^ ^15^	0%^9^ ^,^ ^10^ ^,^ ^17^ ^,^ ^18^	-	-	26.3%^23^	53.8%^23^	IP = 12%^21^ OR = 52%^21^
Infectivity	100%^14^ ^,^ ^15^	100%^10^ ^,^ ^17^ ^,^ ^18^	100%^24^	100%^24^	88.9%^24^	88.9%^24^	IP = 100%^21^ OR = 100%^21^
Morphology of BT	Slender form 70-90%^14^ ^,^ ^15^	Stout form 90%^20^	-	-	-	-	-
Pathology	Acute chagasic phase^15^	Acute chagasic phase; cardiac collagenogenesis; inflammatory infiltrates^17^ ^,^ ^18^	Acute and chronic chagasic phases; cardiomegaly; mild myocarditis^23^ ^,^ ^24^ Cardiac plexus, moderate neurotropism^29^	Acute chagasic phase, severe myocarditis, cardiomegaly; fibrosing chronic cardiopathy, congestive heart failure^9^ ^,^ ^10^ ^,^ ^16^ ^,^ ^23^ ^,^ ^24^ Cardiac plexus, intense neurotropism, inflammation and lesions^29^ Oesophagus and colon, enteric nervous system lesions, inflammatory response with denervation in acute and chronic infections^28^	Moderate myocarditis^16^ ^,^ ^23^ ^,^ ^24^	Severe myocarditis, fibrosing chronic cardiopathy^16^ ^,^ ^23^ ^,^ ^24^ ^,^ ^26^	Oral route acute inflammatory infiltrates in the stomach, duodenum and colon^21^

BT: blood trypomastigote; MT: metacyclic trypomastigote; IP: intraperitoneal; CO: conjuctival; OR: oral.

Differences in the parasitaemia profiles of Berenice strains in mice and dogs infected by different routes with blood or metacyclic trypomastigotes are shown in Table. The parasitaemia profile of the Be-62 strain remained remarkably stable over several occasions (1964 - 1972).^15^ The patent parasitaemia period of Be-62 infection of mice with blood trypomastigotes is shorter (seven days)^14^ ^,^ ^15^ than that of Be-78 (26 days).^16^ ^,^ ^17^ ^,^ ^18^ Be-62 infection exhibits a maximal parasitaemia peak on the 7th day, whereas the Be-78 strain shows two distinct peaks of parasitaemia.^14^ ^,^ ^15^ ^,^ ^16^ ^,^ ^17^ ^,^ ^18^ The virulence of Be-62 is higher than that of Be-78 and the mortality rate is 100% in Be-62 infection,^14^ ^,^ ^15^, whereas the survival rate is 100% in Be-78. The slender and stout trypomastigote forms prevail in Be-62^14^ ^,^ ^15^ and Be-78,^20^ respectively.

Carvalho et al.^21^ compared the effect of the infection route (oral or intraperitoneal) on the parasitaemia and survival rate of mice infected with metacyclic trypomastigotes of Be-78 (Table). There were no significant alterations in the parasitaemia profile between the two routes. However, the mortality rate was higher in the group that was orally infected.

Several detailed follow-up reports have been published on the establishment of the infection and pathogenesis in dogs infected with Berenice strains. Dogs were inoculated through the intraperitoneal or conjunctival route with blood trypomastigotes of Be-62 or Be-78 strains isolated from mice^16^ ^,^ ^20^ ^,^ ^24^ or with metacyclic trypomastigotes of Be-62 or Be-78 from faeces or nymphs of experimentally infected triatomines (*D. maximus* or *T. infestans*)^20^ ^,^ ^23^ ^,^ ^24^ ^,^ ^25^ ^,^ ^26^ (Table). The rates of infectivity were the same (100%) in dogs inoculated via the intraperitoneal route with metacyclic or blood trypomastigotes of both strains.^24^ The inoculation via the conjunctival route resulted in 88.9% of infectivity with metacyclic forms of both strains, while significantly lower rates of infectivity were found in dogs inoculated with blood trypomastigotes of Be-78 and Be-62 (0-11.1%).^24^

The mean of parasitaemia in dogs inoculated, via the intraperitoneal or conjunctival routes, with metacyclic trypomastigotes from Be-62 or Be-78 was higher than of those inoculated with blood trypomastigotes.^24^ ^,^ ^25^ The mean of parasitaemia in dogs infected with metacyclic forms of Be-78 was higher when compared to dogs infected with Be-62.^23^ ^,^ ^24^ ^,^ ^25^ Together, these results support the notion that the conjunctival and oral routes are important factors in the transmission of *T. cruzi* infection and pathogenesis of CD. It has been suggested that *T. cruzi* entry via mucosal sites induces specific responses that activate local immune mechanisms and further modulate regional and systemic immunity.^33^ ^,^ ^34^

The pathogenicity Be-62 and Be-78 strains has been studied using mice and dogs as experimental models^10^ ^,^ ^16^ ^-^ ^18^ ^,^ ^20^ ^,^ ^21^ ^,^ ^23^ ^,^ ^24^ ^,^ ^26^ ^,^ ^28^ ^,^ ^29^ (Table). Infection of dogs with Be-78 induced severe myocarditis and fibrosing chagasic cardiopathy, reproducing experimentally the lesions found in Chagas’ heart disease in humans. In contrast, infection with the Be-62 strain induced mild myocarditis.

The development of the digestive form of CD is characterised by motor abnormalities related to lesions of the myenteric plexus system. Histopathological evaluation of the oesophagus and colon of dogs infected with the Be-78 strain revealed an inflammatory response accompanied by denervation in both acute and chronic phases of infection.^28^ A comparative histopathological study of the cardiac plexus of dogs infected with the Berenice strains showed that inflammation, lesions and tissue parasitism were more severe in animals infected with Be-78 than the Be-62 strain, suggesting that Be-78 is more neurotropic than Be-62.^29^

The susceptibility of Berenice strains to BZN was evaluated in parasite populations (stocks) isolated from outbred dogs chronically infected with Be-62 or Be-78 strains 2-10 years ago.^22^ ^,^ ^30^ ^,^ ^31^ ^,^ ^32^ Variation in susceptibility to BZN was determined in mice as an experimental model. Mice infected with the parental strains Be-62 or Be-78 showed 100% cure after treatment with BZN, whereas Be-62 or Be-78 stocks isolated from long-term infected dogs exhibited 50-90% BZN resistance right after isolation. Furthermore, the maintenance of Be-62 or Be-78 stocks by successive blood passages in mice or *in vitro* culturing can lead to alternation between BZN -resistance and sensitive phenotypes.^22^ ^,^ ^30^ ^,^ ^31^ ^,^ ^32^ The phenotypic analyses indicate that the Be-62 and Be-78 strains are genetically heterogeneous, suggesting intraspecific variation between them.

Genetic profiling of Berenice strains (Be-62 and Be-78)

Molecular methods were successfully introduced for the diagnosis and genotyping of *T. cruzi* in the late 1970s. Isoenzyme and kinetoplast DNA (k-DNA) techniques generated new data and insights into the genetic complexity of trypanosomes. The genetic diversity of Berenice strains was confirmed by analyses of electrophoretic isoenzyme profiles (zymodeme) and kDNA restriction enzyme fingerprints (schizodeme). Lana et al.^19^ determined the zymogram of eight isoenzymes and the kDNA fingerprint of strains Be-62 and Be-78. The experiments were conducted using cultures established in the axenic medium after isolation from mouse haemocultures. Be-62 and Be-78 strains displayed different zymodemes and kDNA fingerprints. The Be-62 zymogram was classified into the zymodeme A for a total of eight enzymes tested, according to the classification of Romanha et al.,^35^ and Carneiro et al.^36^ The same analysis in Be-78 resulted in a mixed zymodeme (A-B) composed of eight isoenzymes, six of which were grouped within the zymodeme A and two others in the zymodeme B. The isolates Be-62 and Be-78 showed different kDNA restriction patterns (zymodemes) after digestion with *Eco*RI.

Lana et al.^19^ proposed alternative hypotheses to explain the genetic diversity observed between Be-62 and Be-78 strains: (1) Berenice was infected once only with triatomines harbouring different *T. cruzi* strains; (2) Berenice acquired a second infection from triatomines carrying another *T. cruzi* strain. Although she denied post-childhood contact with triatomines,^8^ ^,^ ^11^ this hypothesis cannot be excluded; (3) the patient’s immune system selected a different subpopulation of *T. cruzi* in the course of the chronic phase of the infection; (4) the isolation of Be-62 and Be-78 strains by xenodiagnosis with different triatomine species could have selected different populations of *T. cruzi* on each occasion ― *T. infestans* in 1962^8^ and *D. maximus* in 1978.^9^ ^,^ ^10^ These results indicate that genetic variability may occur in subpopulations under different selective pressures depending on the host in which trypanosomes are developing, mammals and triatomines.

In the 1990s, powerful new molecular biology techniques significantly improved the diagnosis and genotyping methods for *T. cruzi*, making them more sensitive and faster with the introduction of amplification steps of the target DNA by polymerase chain reaction (PCR) and DNA sequencing.

Be-62 and Be-78 strains were probed with a large number of genetic markers, 22 isoenzyme loci by multilocus enzyme electrophoresis (MLEE), nine random primers by random amplification polymorphic DNA (RAPD) and seven microsatellite loci by simple sequence repeat anchored PCR (SSR-PCR).^36^ Parasites were maintained throughout successive blood passages in mice, isolated by haemoculture and cultivated in axenic culture medium. Comparison of the results confirmed the presence of genetic differences between Be-62 and Be-78 strains, but to a lesser extent than previously reported.^19^ Among the 22 isoenzyme loci tested by MLEE, only two showed different profiles between the Be-62 and Be-78 strains. The authors classified the Be-62 and Be-78 strains into the zymodeme A. Of the nine primers tested in the RAPD assay, only three were able to differentiate the Be-62 and Be-78 strains from each other. Parental strains Be-62 and Be-78 showed different profiles in the SSR-PCR assay.

Cruz et al.^37^ also investigated Berenice strains’ genetic structure by typing microsatellite loci. The parental strains were isolated from mouse haemocultures and cultivated in an axenic medium (LIT). The clones were then isolated by limiting dilution and plating on a solid agar-LIT medium. The clones of Be-62 and Be-78 strains presented a genetic profile equivalent to their respective parental strains when seven microsatellite *loci* were tested, suggesting that Be-62 and Be-78 were monoclonal populations. However, they noticed that the absence of subpopulations in the two strains would also be due to the method’s sensitivity, and more sensitive methods would be necessary to validate this hypothesis.^37^ Assuming that the patient Berenice was infected only once, the host’s immune system played a crucial role in controlling *T. cruzi* in long-term infections.^37^ Both strains, Be-62 and Be-78, were classified in the phylogenetic lineage DTU - Tc II, indicating they are phylogenetically related.^37^

The adaptation of Be-62 and Be-78 strains to the canine experimental model^16^ ^,^ ^20^ ^,^ ^23^ ^-^ ^29^ provided new insights into the role of the genetic structure of *T. cruzi* in long-term infections in vertebrate hosts. Veloso et al.^14^ compared the biological and genetic characteristics of the Be-78 strain in different hosts, including dogs and mice. Be-78 parasites were isolated from dogs in the chronic indeterminate and cardiac forms, two and seven years after the infection. Immediately after isolation, the parasites were re-inoculated and maintained in mice by serial blood passages. After the first and 25th passages, the parasites were isolated by haemoculture and cultivated in LIT to evaluate the multiplication of epimastigotes and metacyclogenesis. The profile and percentage of parasitaemia of Be-78 isolates in mice ranged from parasitaemia curves with defined peaks accompanied by high parasitaemia in some Be-78 isolates to irregular peaks with low parasitaemia in others. The histopathological analysis showed variable intensity (moderate to severe) of inflammatory infiltrates in the cardiac tissue.

The genetic variability of Be-78 parasites isolated from dogs after mice’s 1st and 25th passages was probed with five polymorphic isoenzyme *locus* markers and five random primers by RAPD.^35^ An interesting finding was the detection of different zymodemes in the same isolate. After the 1st passage, the isolates shared the same isoenzyme profile with the parental strain, while others displayed a different profile. According to the authors,^14^ the presence of three zymodemes in different proportions in most isolates suggests the existence of at least three subpopulations within the parental Be-78 strain. RAPD confirmed the results obtained from zymodeme analysis.

The results suggested that the Be-78 is a multiclonal strain.^14^ This conclusion is based on more than two alleles for the markers used in the analysis. However, the genetic variability among the parasites present in the host could also be explained by aneuploidy.^37^ In this case, the host would be infected by a monoclonal population built of genetically different subpopulations that would have evolved from a major population by aneuploidy, that is, changes in the number of one or more chromosomes (chromosomal aneuploidy) or duplication of a segment of a given chromosome (segmental aneuploidy).

To answer this question, Valadares et al.^38^ devised an exquisite experiment that allowed the genotyping of an individual parasite. The authors adapted a flow cytometry population protocol (FACS Single Cell Sorting), initially proposed for analysing a single sperm, to isolate a single epimastigote. The isolates obtained from a dog experimentally infected with the Be-78 strain were inoculated into mice and re-isolated by haemoculture after the 1st (Be-78.1B) and 25th (Be-78.25B) passages. Afterwards, the parasites were cultivated in LIT medium, and about 1 million epimastigotes were collected, fixed and separated by flow cytometry. Several controls were carried out to ensure the isolation and cultivation of a single parasite. The genotyping of epimastigotes isolated by FACS was performed using four microsatellite loci, resulting in the identification of three different subpopulations. One subpopulation displayed the same genotype of the parental B-78 strain, while the other subpopulation displayed the genotype of the B-62 strain, and a third subpopulation presented a new genotype. The combination of alleles of the four microsatellites, equivalent to a given genotype, was identified in individualised cells of the Be-78.1B and Be-78.25B isolates, confirming that the Be-78 strain has multiclonal structure and that the genotypes were not the result of aneuploidy events in a monoclonal strain. As previously discussed, the patient Berenice could have been infected with triatomines carrying mixed *T. cruzi* populations.^8^ ^,^ ^11^ ^,^ ^19^ Then the Be-62 and Be-78 strains would have been present since the moment of infection, and their predominance might be related to the mammalian host defence system.

Nogueira-Paiva et al.^39^ investigated whether the evolution of the pathogenesis of CD is related to the selection by the vertebrate host of subpopulations within a *T. cruzi* multiclonal strain. Swiss outbred mice were inoculated with Be-62 or Be-78 strain, and the kDNA profile was determined by kDNA typing via LSSP-PCR of parasites isolated from blood samples collected after the 3rd, 6th, and 12th months of inoculation. The Be-62 and Be-78 strains showed different kDNA profiles by LSSP-PCR,^39^ confirming the heterogeneity between these two strains as demonstrated by other methodologies. Most subpopulations isolated from Swiss outbred mice infected with the Be-78 strain were genetically correlated with the parental strain Be-78. They also identified a subpopulation that was significantly distinct from the parental strain Be-78. Different kDNA profiles were detected by LSSP-PCR among isolates from the same animal at other times of infection. However, it was impossible to associate the genetic alterations with biological characteristics. The results suggested that a subpopulation may have been favoured at a given time and be replaced by another population throughout the development of the chronic *T. cruzi* infection.

Recently, a high-quality whole genome assembly of the Berenice strain has been generated by Díaz-Viraqué et al.^40^ using a blood sample from a mouse infected with the strain Be-62 isolated in 1962 from the patient Berenice. This isolate has been cryopreserved in liquid nitrogen and thawed to be used as the source of DNA. Sequence data was obtained from two next-generation sequencing platforms, Illumina/Solexa, and the third-generation MinION Oxford Nanopore, resulting in an excellent assembly of the Berenice genome with a size of ~40 Mb. The assembly comprises 13,763 genes and 596 pseudogenes, distributed 13,307 protein genes, 46 rRNA genes, 69 tRNA genes, and 341 other RNA genes (snRNA, snoRNA, etc.). This represents a chromosome-scale genome sequencing, assembly, and annotation of the Berenice strain (lineage TcII).

Be-62 scaffolds share regions of similarity with homologous chromosomes from different *T. cruzi* strains (Fig. 2). For example, the scaffolds JABDHM010000009.1 (530,098 bp) and JABDHM010000002.1 (726,303 bp) share 97.0-99.1% similarity with syntenic regions of chromosomes TcChr37-S (1,361,062 bp- haplotype Esmeraldo-like) and TcChr37-P (1,361,062 bp- haplotype non-Esmeraldo like) of clone CL Brener (TcVI, hybrid strain), 97.0-97-99% similarity with syntenic regions of chromosomes TcBrA4_Chr4 (1,676,910 bp) of Brazil strain (TcI) and TcYC6_Chr4 (1,578,048 bp) of Y strain (TcII).

**Fig. 2:**
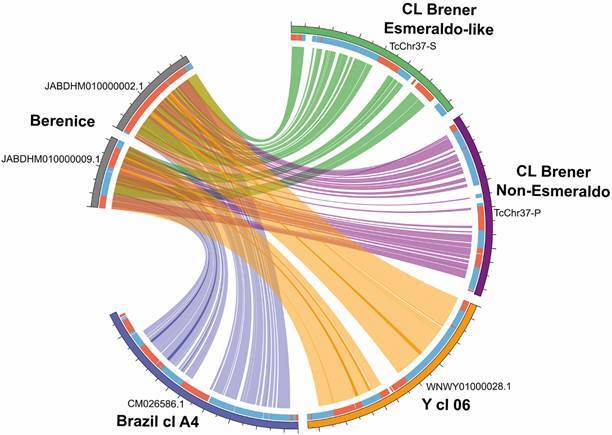
genomic synteny: circos plot showing chromosome 37 syntenic regions from different *Trypanosoma cruzi* lineages (Tc). The outer coloured (grey, green, purple, orange, and blue) track represents the chromosome 37 homolog loci from *T. cruzi* strains Berenice (Be-62, TcII); CL Brener Esmeraldo and Non-Esmeraldo Like (hybrid genome assembly, TcVI); Y cl 06 (TcII) and Brazil cl A4 (TcI), respectively. The inner track, painted blue and red, shows forward or reverse-oriented polycistronic gene blocks, respectively. Inner ribbons represent conserved regions detected using BLASTn between Berenice and CL Brener (Esmeraldo and Non-Esmeraldo haplotypes), Brazil clone A4 and Y clone 06 *T. cruzi* strains. Ribbon colours match the respective strain outer strain colour. Syntenic relationships were determined using the BLASTn algorithm, e-value of 1e-5 and ≥ 10,000 bp coverage cutoffs using Berenice strain (scaffolds JABDHM010000002.1 and JABDHM010000009.1) as queries against the draft genomes of Brazil cl A4 (CM026586.1), Y cl 6 (WNWY01000028.1) and CL Brener Esmeraldo-like and non Esmeraldo-like haplotypes (TcChr37-S and P). The best hits matching a target chromosome or large scaffold were selected, and results were plotted using a Circos plot diagram.

The remarkable level of conservation in the syntenic regions of Be-62 strain after decades-long persistence in the patient Berenice is in agreement with the hypothesis that *T. cruzi* genetic diversity is driven by the evolution of multigene families encoding target antigens of anti-parasite immune responses.^41^ ^,^ ^42^ Interestingly, the multigene families (TS, trans-sialidases; MASP-Mucin Associated Surface Proteins and mucins) of *T. cruzi* are located in non-syntenic disruptive compartments of the genome.^43^ Recent analyses have demonstrated greater expansion and complexity for *T. cruzi* multigene families involved in the infection, virulence and pathogenicity compared to other trypanosome species.^43^ ^,^ ^44^ *T. cruzi* multigene families are species- or clade-specific genes located in a genomic region under positive selection towards the adaptation and survival of the parasite in the mammalian host. The non-syntenic disruptive compartments have been considered a recent genome region.^43^

As previously discussed, Berenice strain has carried substantial genetic diversity, which could be acquired in a single bite with a triatomine carrying multiple *T. cruzi* strains or a result of multiple bites of triatomines carrying different strains. The sequencing of the Be-78 genome and its comparison with the Be-62 genome may provide information about the origin of these strains. Perhaps Be-78 was a subpopulation of the Be-62 strain, resulting from the ongoing adaptation of the parasite in patient Berenice. The presence of genetically distinct parasites could drive the evolution of parasite traits, such as virulence, drug resistance and fitness, resulting from competition between different Berenice haplotypes within the host. Single-cell NGS sequencing technology appears to be the method of choice for answering the questions raised in the pioneering works of Valadares et al.^38^ and Nogueira-Paiva et al.^39^ on the genetic structure of Berenice strains. While single-cell genomics can resolve genomic diversity, single-cell transcriptomics identifies functional differences and hidden heterogeneity within parasite populations by capturing gene expression profiles often overlooked in comparative genomic approaches. This combined approach has the potential to integrate parasite genetic diversity to the transcriptional signatures associated with parasite adaptation, virulence and drug resistance, offering a deeper understanding of host-parasite interactions.

What are the next steps regarding the contribution of patient Berenice to the natural history of CD? What are the factors influencing host-parasite interactions? As previously discussed, the post-genomic era has already begun in the study of parasite infections. Single-cell omics technology has been applied to determine the genetic diversity within infections caused by protozoan parasites, such as *Leishmania* and *Plasmodium*. It allows us to estimate the pre-existing genetic variants within an infection and the contribution of each type of variation.^45^ ^,^ ^46^

Approximately 115 years after the discovery of CD, the Berenice strain remains available to researchers of CD and other protozoan parasitic infections. In conclusion, we can still learn from the Berenice strain of *T. cruzi*.
